# Eliciting a User’s Preferences by the Self-Disclosure of Socially Assistive Robots in Local Households of Older Adults to Facilitate Verbal Human–Robot Interaction

**DOI:** 10.3390/ijerph191811319

**Published:** 2022-09-08

**Authors:** Toshiharu Igarashi, Misato Nihei, Takenobu Inoue, Ikuko Sugawara, Minoru Kamata

**Affiliations:** 1 Department of Human and Engineered Environmental Studies, The University of Tokyo, Kashiwanoha 5-1-5, Kashiwa 277-8563, Chiba, Japan; 2 Institute of Gerontology, The University of Tokyo, 3-1, Hongo 7-Chome, Bunkyo-ku 113-8654, Tokyo, Japan; 3 Research Institute of National Rehabilitation Center for the Persons with Disabilities, 1, Namiki 4-Chome, Tokorozawa 359-8555, Saitama, Japan; 4 Bunri University of Hospitality, 311-1, Kashiwabara-Shinden, Sayama 350-1336, Saitama, Japan

**Keywords:** socially assistive robots, self-disclosure, human–robot interaction

## Abstract

To realize a society in which older adults can live independently in their homes and familiar environments for as long as possible, their lives can be supported by providing appropriate technology. In this case, a new intervention for older people using socially assistive robots (SARs) is proposed; however, previous research has demonstrated that individual differences exist in the use and response to SAR interventions, and it has also been reported that SARs are not used by users in some cases. Therefore, in this study, we developed a self-disclosure function to promote continuous interaction with robots, using a Japanese corpus and self-disclosure items. In this study, we defined the specific requirements and functions of self-disclosure in SARs and developed ten non-arbitrary speech scripts from the field of social psychology using a Japanese corpus and self-disclosure items. To evaluate the effect of self-disclosure in SARs, an SAR was introduced to each household for 20 days, with the consent of seven community-dwelling older adults. Based on the recorded voice interaction data, we analyzed how the number, total time, and quality of verbal interactions changed with the SAR’s self-disclosure. Furthermore, we conducted group interviews with the participants and received positive comments regarding the robot’s self-disclosure. Some participants considered the specific personality of the SAR by accumulating its behavioral characteristics. As a consequence, these results indicate that the robot’s self-disclosure feature is effective in significantly increasing the quantity and quality of verbal interactions with older adults.

## 1. Introduction

### 1.1. Changing Lifestyles of the Older Adults in Japan

Japan is currently a super-aged society with an aging rate of 28.9%. According to the White Paper on Aging Society for the year 2022, the total population continues to decline while the population aged 65 and over continues to increase. It is expected that the proportion of older adults will continue to increase at a constant rate, reaching 38.4% by 2065 [1]. Japan has the highest aging rate in the world, and it is expected to remain high in the future.

As a national policy, the Ministry of Health, Labour and Welfare (MHLW) has presented the “Healthy Life Expectancy Extension Plan” to promote the extension of healthy life expectancy and to ensure the sustainability of social security by reviewing benefits and burdens [2]. Although it is known that prevention through the formation of healthy lifestyles and early detection and intervention of diseases and illnesses will lead to a reduction in the increase in medical and long-term care costs, long-term and continuous support is necessary for the formation of healthy lifestyles and disease prevention, including dementia. For this reason, recent welfare programs for older adults in Japan have been promoting the development of a community-based comprehensive care system environment that facilitates access to home nursing, home care, and day care services.

However, the living arrangements of older adults have changed in Japan, and while in the past two or three households living together were the norm, in recent years the number of single-person households and older couple households has been increasing. In fact, the percentage of solitary/older couple households in the total number of households containing persons aged 65 and over reached a record high of 60.1%. One of the problems resulting from the increase in the number of single-person households and older couple households is the decrease in opportunities for conversation within the household. According to the results of the Cabinet Office’s “Survey of Elderly People’s Views on Housing and Living Environment,” only about 65% of older people living alone engage in conversation every day, which is clearly lower than the more than 90% of older couple households and households with children living together [1]. The 2010 Tokyo Metropolitan Basic Survey on Social Welfare and Health revealed that about half of older adults who live with their families are alone at home during the day at least one day a week [2]. Of these, another half reported being alone at home four or more days a week, suggesting that there are many older people who live with their families but frequently find themselves in a state that is virtually the same as living alone, and that although they speak with their families every day, they rarely speak with them during the day. It is thought that there are many older people who have conversations every day but rarely talk during the daytime.

In addition, as the number of single-person households and older couple households increases, it becomes difficult to provide individualized support. Therefore, prevention through the formation of healthy lifestyles is important so that they can live independently and autonomously at home for as long as possible. However, due to the system of a comprehensive care system and the lack of human resources, it is necessary to provide lifestyle support with appropriate technology in order to realize such a society.

To realize the vision of a society in which older adults can live independently in their homes and familiar environments for as long as possible, their lives can be supported by providing appropriate technology. To this end, a new intervention for older people using socially assistive robots (SARs) has been proposed [3]. Since the 2000s, researchers have extensively focused on the use of SAR, and there has been a significant interest in supporting disabled people. Previous intervention studies have measured and reported the effectiveness of implementation in supporting older people if the SAR is used properly by the user. Because it is expected to be effective for older and depressed people [4], the creation of a continuous relationship with an SAR can improve the safety and quality of the life of users.

However, previous research demonstrated that individual differences exist in the use of and response to SAR interventions, and it has been reported that SARs are not used by users in some cases [5]. In a systematic review by Tijs et al. [6], four factors related to SARs were described as the main factors affecting utilization: (a) roles of the SAR, (b) appearance of the SAR, (c) normative and/or ethical issues regarding the use of SARs in aged care, and (d) interaction between older adults and the SAR. As for (a) roles, (b) appearance, and (c) normative/ethical issues on the use of SARs in aged care, these areas and topics have been intensively researched [7,8]. However, there is a paucity of information on (d) the interaction between SARs and older adults. Takama et al. indicated that new features and attractions, not simply high functionality, are necessary for robots to be recognized as partners by co-living users in their households [9].

Thus, SARs have not yet reached the point that they can be introduced to target older adults living in the community; therefore, the development of their functionality to increase interactions is an important research topic.

### 1.2. The Position and Contribution of This Study

Although self-disclosure by robots is a promising research topic, three major issues remain unresolved.

First, a systematic method for self-disclosure by robots has not yet been completely determined. As each study conducted a different intervention with the concept of self-disclosure in the HRI research fields, it is difficult to evaluate the self-disclosure effects of each study. Therefore, it is necessary to define these requirements and develop a specific, non-arbitrary function for self-disclosure using SAR.

Second, the effect of self-disclosure by robots is mainly qualitative, such as through questionnaires, and the content of conversations with robots has rarely been evaluated quantitatively. It is not possible to quantitatively evaluate self-disclosure by robots without acquiring and analyzing the speech data of all interactions for future social implementation, even though doing so raises significant difficulties in ethical regulations and privacy. Furthermore, it is not yet clear how the participants perceived the robot’s self-disclosure or how it was involved in the interaction.

Third, these studies were mainly implemented in laboratories or rooms for experimentation, which means that limited temporary results were obtained during a few hours of experimentation. Mutual self-disclosure by SARs and older adults may be inhibited in a particular environment for participants where the experimenter is present. Considering the concept and role of SARs rooted in the lives of users, experimental data in places where participants usually live are preferable for future social implementation, although there are significant difficulties in ethical and privacy issues.

Therefore, this study aims to create a method of self-disclosure by the robot from the perspective of social psychology and to analyze its impact on the user in terms of the number of interactions and the amount of conversation per interaction. The study will also analyze qualitatively not only the quantity of conversations, but also how the content of the utterances was transformed. Furthermore, this study will be conducted in cooperation with the Kamakura Living Lab, which will provide results not from a specific experimental setting, such as a university, but from daily use by older people living in the community.

## 2. Related Works

### 2.1. Self-Disclosure in Human Interactions

The question of how humans can improve communication has long been discussed in the field of social psychology. Although various studies have focused on communication with each other, self-disclosure, studied by Jourard et al. since the late 1960s, has the effect of building mutual acceptance and trust relationships with others. Self-disclosure is defined as a process in person–person communication in which an individual reveals information about themselves to another person [10]. The literature on self-disclosure in human relationships is extensive, and comes primarily from psychology, communication sciences, and sociology. Frattaroli conducted a meta-analysis of 146 randomized studies and found that self-disclosure had a positive effect on the well-being of those who disclosed their thoughts [11]. Pennebaker and Beall reported that participants who disclosed their thoughts and feelings about traumatic events over several weeks showed an objectively improved health status [12]. In connection with this, self-disclosure can trigger social support [13], which is classified into four aspects: emotional, material, informational, and appraisal support [14]. Disclosing one’s own troubles to others provides important information for these types of support.

Empirical research on self-disclosure has primarily focused on the link between self-disclosure and liking for others. A comprehensive meta-analysis including 74 studies by Collins and Miller [15] revealed interesting findings regarding self-disclosure. People who make a large amount of self-disclosure tend to be more liked than those who disclose at lower levels, and people who disclose themselves may reciprocally like others as a result of self-disclosure in the first place. This research shows that self-disclosure occurs first in person–person communication, and favors from others follow as a result of self-disclosure. Moreover, it has been reported that technology facilitate human self-disclosure. Ikeda et al. used a method to promote communication between speakers who had never previously met via communication using a system that suggests topics for self-disclosure by arranging the displayed information [16].

Thus, previous research on self-disclosure has found that not only does self-disclosure positively affect the health and well-being of those who disclose themselves, but it also improves liking for those who self-disclose, and that self-disclosure can be facilitated by certain technical methods.

### 2.2. Effectiveness of SAR

An SAR is defined as “a robot that provides assistance to users by social mutual interaction” [17]. Hence, interpersonal methods to enhance communication, such as self-disclosure, can be applied to human–robot interaction (HRI) and are considered effective. Some studies have demonstrated that robots and anthropomorphic agents are persuasive to people [18]. Yamamoto also states that anthropomorphic shapes have the advantage that they are easy to talk to and are easily forgiven by the user when misperception or misoperation occurs [19]. It also states that the robot is effective as an anthropomorphic information terminal that works on the person from the terminal without the user’s operation. Thus, SAR has various advantages in intervening with older adults.

Since the 2000s, research utilizing SAR has been in full swing, and studies have been conducted to verify the effectiveness of SAR in supporting the disabled and older adults. In overseas cases, SAR has been demonstrated to be effective in improving the lives of older adults, depressed participants, and children with developmental disabilities. According to Banks et al. (2008) and Shibata, Wada, Saito and Tanie (2001), robots that imitate animals in particular have the same beneficial effects as live animals, and these robots can elicit the same level of attachment as live dogs [20,21].

In Japan, a trend is emerging to utilize SAR as a monitoring system in older care facilities to actively address the existing shortage of caregivers and to work toward care prevention. According to a study by Ninomiya (2015), a chronological change has been reported that the use of PALRO has increased smiles, enriched facial expressions, and facilitated communication [22]. In recent years, pet-like robots without verbal communication, such as LOVOT [23] and Aibo [24], have also been developed and marketed in Japan; in 2013, the first two randomized controlled trials (RCTs) using PALRO with dementia and older participants were reported and both studies demonstrated that social robots may have beneficial effects on psychological well-being [25,26].

### 2.3. HRI and Self-Disclosure

With the arrival of an aging society, expectations are rising for the realization of service robots that can assist humans in their living environment, but the problem is how to make robots recognized as partners. In a study by Takama et al., it was also stated that in order for robots not to become bored and to gain a sense of trust when introduced to each household, merely having high functionality is not enough; it is necessary to devise ways to have robots recognized as partners who share living spaces [9].

Against this background, the assignment of personality to SAR has been attracting attention in recent years. Nakagawa et al. compared the personalities of SARs on the continuation of healthy behaviors with those of directive and submissive personalities, and demonstrated that submissive personalities are more effective in making people take healthy behaviors [27]. This indicates that the imposition of personality may be able to promote the effects of the originally intended intervention (e.g., continued exercise). Ogawa et al. also demonstrated that even though the agent on the display is an artificial entity, the user applies the other person model to the agent and perceives its personality through the sentences it reads and its appearance [28]. This raises the question that even if a personality is given to an inorganic entity such as a robot, the personality of the robot will not be recognized by the user, because, at least in Japanese, even if a personality is given to an inorganic object such as a robot, the cognitive process in which the user applies the other person model to it will not be impaired. The question is that, at least in Japanese, the cognitive process of applying the model to an inorganic object, a robot, is not impaired by assigning a personality to it.

Kang and Gratch [29] investigated the effects on the perceptions and behaviors of human interaction partners when virtual counselor agents disclosed themselves to humans; in the experiment, the agents revealed information about themselves and then asked participants 10 questions. The results of the questionnaire demonstrated that participants’ empathy and social attraction toward virtual counselor agents were increased by their self-disclosure. In addition, Kumazaki et al. reported that communication with visually simple robots tends to promote human self-disclosure of negative topics more than does human-to-human communication [30]. Moreover, in the context of social mediator robots operated by humans, Noguchi et al. demonstrated that self-disclosure robots were liked more by older adults who had experienced loss when the robot disclosed their experience of loss [31].

To summarize the HRI research on self-disclosure to date, these studies suggest that personal robots facilitate self-disclosure in the positive and negative aspects of older adults, which contributes to the favor of robots by older people. However, these are experiments conducted on the spot between the experimenter and the participants, and are only verified in an environment where the experimenter and the experiment participants are present and communication itself is enforced. In this case, even if there is a temporary effect, it is not clear whether it will continue to be used. In order to solve the utilization issues raised in the introduction, it is necessary to develop functions that promote interaction itself.

## 3. Self-Disclosure by SARS

### 3.1. Requirement Functions

There are three required functions related to self-disclosure below:

1. Self-disclosure function:
  Reveal information about SARs to users.
2. Voice synthesis function:
  Conversion of text data into speech using a voice synthesis system.
3. Voice recording function:
  Record feedback from the user through a built-in microphone.

For the self-disclosure function, different self-disclosed speech scripts were required to analyze the effect of self-disclosure by SARs. To prevent arbitrary speech scripts from being composed, they were constructed using the following procedure: defining self-disclosure items, exclusions of speech scripts, and adjusting speech script structure.

### 3.2. Defining Self-Disclosure Items

As the basis of self-disclosed speech scripts, we used a Japanese blog corpus, the Kyoto University Analyzed Blog Corpus (KNB), developed by Hashimoto et al. [32] (Table 1). The KNB corpus consists of 249 articles on four themes (sightseeing in Kyoto, mobile phones, sports, and gourmet dining), and has been used as a speech script for agents. By contrast, the KNB corpus contains a variety of texts, some of which do not include self-disclosure. Therefore, we used the Jourard Self-Disclosure Questionnaire [33] as a criterion for self-disclosure in the context of social psychology. In this study, a statement in the KNB corpus that meets the Jourard Self-Disclosure Questionnaire item is defined as a self-disclosure speech script.

### 3.3. Exclusions of Speech Scripts

The Jourard Self-Disclosure Questionnaire consists of general interpersonal items that are assumed to be communicative: (a) items that are unnatural if spoken by the robot, (b) items not relevant to older people, and (c) items not natural for Japanese people. Therefore, we examined and excluded items on political and religious factors, periodicity, specific locations, financial circumstances (such as personal assets and debts), sexual orientation, and gender. After exclusionary work, only ten articles from the KNB corpus met the requirements of the Jourard Self-Disclosure Questionnaire items.

### 3.4. Adjusting the Speech Script Structure

**Figure 1:**
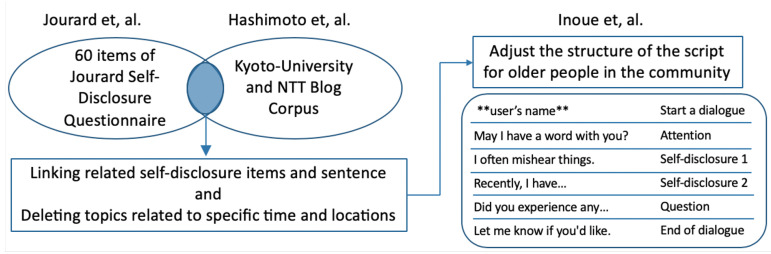
The process of creating a self-disclosure script in this research [32,33,34].

In this study, SAR intervention in each household was assumed. To reduce the possibility of the robot’s speech going unnoticed by users in their homes, the structure of the speech script was adjusted according to Inoue et al.’s research [34]. The script consisted of five steps: (1) starting a dialogue by calling the user’s name, (2) calling the user’s attention, (3) self-disclosure by robots, (4) asking the user questions, and (5) confirming that the user heard and prompting responses. Figure 1 illustrates the procedure for creating a self-disclosure script for this study.

## 4. Intervention Experiments

### 4.1. Participants

The participants were seven older adults (three men and four women) aged > 60 years living in Kamakura City (Table 2). The selection procedure involved first holding a workshop in the target area and then selecting individuals based on their age and gender. Additionally, the interaction was performed verbally; thus, the participants had to maintain cognitive function to be able to hold a conversation. Therefore, the cognitive function of all participants was examined using the Mini-Mental State Examination (MMSE), which is a widely used cognitive function test among older adults. The MMSE is a 30-point scale; a score of 28 or more on the MMSE indicates cognitive health; a score below 27 is indicative of mild cognitive impairment (MCI) (sensitivity (45–60%; specificity, 65–90%); and a score of 23 or less indicates suspicion of dementia (sensitivity 81%, specificity 89%) [35,36,37,38]. As a result of the MMSE, three participants were considered healthy older adults and four were suspected to have mild cognitive impairment.

### 4.2. Equipment

**Figure 2:**
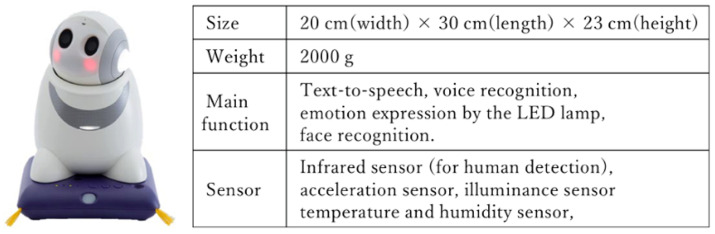
Size, weight, main function, and equipped sensor of Papero-i.

In this study, we used Papero-i from NEC Platforms, as it satisfied the required functions, which are a text-to-speech (TTS) function to realize conversations with users and a voice recording function to collect interaction data in the intervention [39]. Papero-i can be connected to a wireless communication network, and it can transmit self-disclosed speech scripts from the server and store the recorded user’s speech to the server. In addition, as a basic feature, Papero-i has a scheduling support function that informs users of their schedule at an arbitrary time. The specifications of the Papero-i are shown in Figure 2.

### 4.3. Intervention Methods

**Location:** Each participant’s household in the community;
**Period:** 20 days (10 days without self-disclosure and 10 days with self-disclosure);
**The basic role of Papero-i:** Scheduling information support for users.

Because Papero-i has a basic scheduling support function, we interviewed the participants in advance about their weekly and daily schedules and set up scheduling support 10 times per day, which means a total of 100 times for 10 days per participant. Although the content of support depended on the use schedule, it mainly included notification of hospital visits or day care times, and support for the introduction of daily medication and exercises for users. The intervention lasted for 20 days in the form of a pre- and post-controlled trial. While Papero-i provided only schedule support in the first 10 days of intervention, Papero-i disclosed itself using self-disclosure speech scripts created once a day in addition to basic schedule support in the next 10 days of intervention. Before the experiment, all the participants were briefed on the basic role of Papero-i and how to use the robots. We also confirmed that (a) the schedule information support worked as planned, (b) there were no audio problems with SARs, and (c) SARs were set up on a table in the living room. This experiment was approved by the University of Tokyo after ethical review.

### 4.4. Evaluation

#### 4.4.1. Methods and Measured Items

In the experiment, voice interaction data with older adults and SARs were recorded, and (a) the number of interactions with the participants and (b) the total interaction time between the first 10 days and next 10 days were evaluated. In addition, in this study, all recorded audio data were converted to text data, and sentiment analysis was performed to observe emotional changes in the user’s speech. Furthermore, since the effect of the intervention cannot be completely grasped via quantitative evaluation alone, group interviews were conducted to determine how self-disclosure was perceived by the participants.

#### 4.4.2. Results

During the 20 days of intervention, (a) the mean number of interactions was 5.7 (SD = 3.9) in the first 10 days; however, it increased to 10.6 (SD = 1.6) in the next 10 days with SAR’s self-disclosure. Although the number of interactions increased for the six participants, it remained unchanged for one participant. As for (b), the mean of total interaction time was 112.6 s (SD = 97.0) in the first 10 days, though increasing to 255.7 s (SD = 58.9) in the next 10 days with SAR’s self-disclosure. The interaction time with SARs increased for all participants.

#### 4.4.3. ANALYSIS

**Figure 3:**
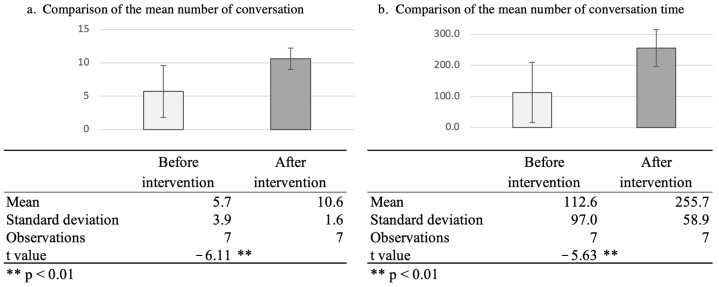
Comparison of the average number of conversations and duration of conversations between robots and users before and after the self-disclosure intervention (10 days each).

To test whether there was a statistically significant difference with or without self-disclosure in the intervention, *t*-tests were conducted for the mean number of interactions and total interaction time in ten days for each older household. There were significant differences in the number and duration of the interactions before and after the intervention. (*p* < 0.01). Figure 3 shows the number of interactions and interaction time during the intervention and the results of the *t*-test.

## 5. Human Trait Analysis

Because individual differences are particularly important when social robots are used in mental healthcare [40], the effect of self-disclosure by SAR should be assessed according to the characteristics of individual users (cognitive function and personality traits).

### 5.1. Impact of Participants’ Cognitive Decline on Self-Disclosure Interaction

Based on the MMSE, three of the seven participants were judged to be cognitively healthy and four were suspected to have mild cognitive impairment. There was a 1.5-year difference in age between the three healthy older group and four cognitively impaired groups, indicating a similar age range. The mean number of interactions increased from 5.3 to 11.3 in the healthy older group, while the mean number of interactions increased from 6.3 to 9.7 in the cognitively impaired group. The effect of the self-disclosure intervention was higher in the healthy older group when viewed as a percentage increase; however, a *t*-test of means, assuming two separate populations, did not reveal a significant difference (*p* > 0.05). Comparing the total interaction time, the healthy older group increased by 150.8 s from 125.0 s to 270.8 s on average, while the cognitively impaired group increased by 139.7 s, from 96.0 s to 235.7 s, on average. The effect of the intervention was higher in the healthy older group when viewed as a percentage increase; however, a *t*-test of the means, assuming two separate populations, did not reveal a significant difference (*p* > 0.05).

### 5.2. Impact of Participants’ Personality on Self-Disclosure Interaction

Recent theories on personality traits share a framework consisting of five components: extraversion, agreeableness, diligence, neuroticism, and openness. In previous research, it is well known that matching robot personality affect has a positive influence on the humans’ task performance [41,42,43,44,45]. The extroversion dimension of the Big Five personality model [46] has often been the focus of HRI research and includes aggressiveness or sociability. Some studies have reported that a person was attracted more to others who were matched in personality than to those who were mismatched [47,48]. Furthermore, Ronen demonstrated that mutual self-disclosure increases among similar extroverted dyads [49]. On the other hand, older adults with lower self-esteem, which is known to be negatively correlated with neuroticism in the Big Five personality model, tends to become less self-disclosure [50,51]. The effect of SARs’ self-disclosure must be confirmed by understanding the participants’ personality traits in the experiment. In this study, we examined each participant’s personality traits using the TIPI-J (Japanese version of the TIPI), and analyzed whether there was participant bias in the effect of self-disclosure in the intervention [52].

#### 5.2.1. Analysis of Extraversion

**Figure 4:**
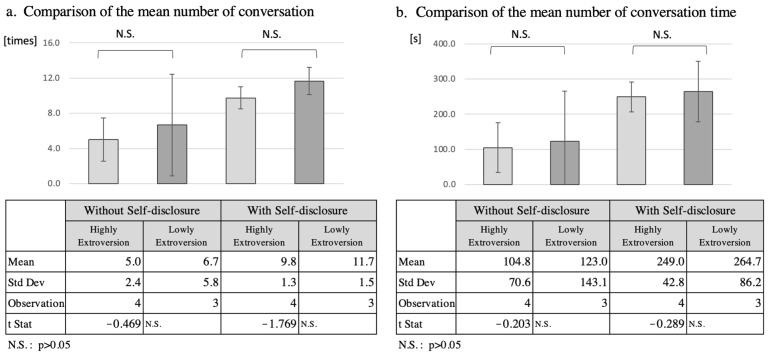
Comparison of number of conversation and speech duration according to high and low extroversion.

Since four of the seven participants had an extroversion score of 4.5 or more, and three participants scored less than 4.5, these two groups were compared as the high extroversion group and the low extroversion group, respectively (Figure 4). The mean number of interactions increased by 4.8 in the high extroversion group from 5.0 to 9.8, while in the low extroversion group it increased by 5.0, from 6.7 to 11.7. The total interaction time increased by 144.2 s from 104.8 s to 249.0 s in the high extroversion group, while in the low extroversion group it increased by 141.7 s from 123.0 s to 264.7 s. However, *t*-tests of the mean number of interactions and total interaction time for the two separate groups did not reveal a significant difference (*p* > 0.05).

#### 5.2.2. Analysis of Agreeableness

**Figure 5:**
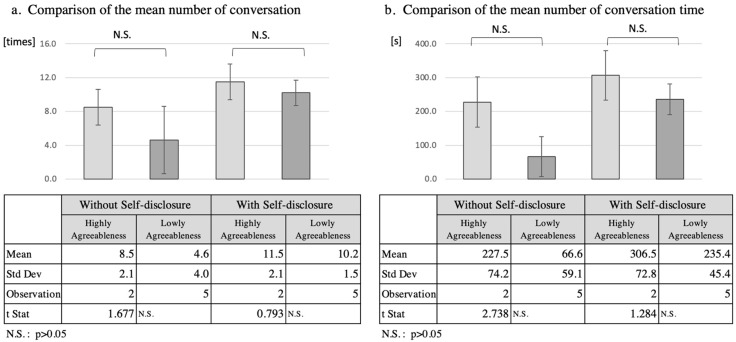
Comparison of number of conversation and speech duration according to high and low Agreeableness.

Since two of the seven participants had a coordination score of ≥6.5, and five participants scored less than 6.5, these two groups were compared as high- and low-agreeable groups, respectively (Figure 5). The mean number of interactions increased by 4.8 In the high-extroversion group from 5.0 to 9.8, while in the mean number of interactions increased by 5.0, from 6.7 11.7. The total interaction time increased by 144.2 s from 104.8 s to 249.0 s in the high extroversion group, while in the low extroversion group it increased by 141.7 s from 123.0 s to 264.7 s. However, *t*-tests of the mean number of interactions and total interaction time for the two separate groups did not reveal a significant difference (*p* > 0.05).

#### 5.2.3. Analysis of Diligence

**Figure 6:**
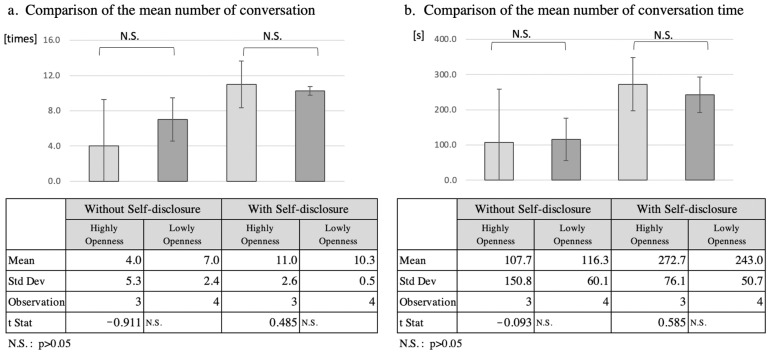
Comparison of number of conversation and speech duration according to high and low conscientiousness.

Since two of the seven participants had diligence scores of ≥5.0, and five participants had diligence scores below 5.0, these two groups were compared as the high-diligence group and the low-diligence group, respectively(Figure 6). In the high-diligence group, the mean number of interactions increased by 1.5 from 8.5 to 10.0, while in the low-diligence group it increased by 6.2 from 4.6 to 10.8. The total interaction time increased by 89.5 s from 132.0 s to 221.5 s in the high-diligence group, while in the low-diligence group it increased by 164.6 s from 104.8 s to 269.4 s. However, *t*-tests of the mean number of interactions and total interaction time for the two separate groups did not reveal a significant difference (*p* > 0.05).

#### 5.2.4. Analysis of Neurotic Tendencies

**Figure 7:**
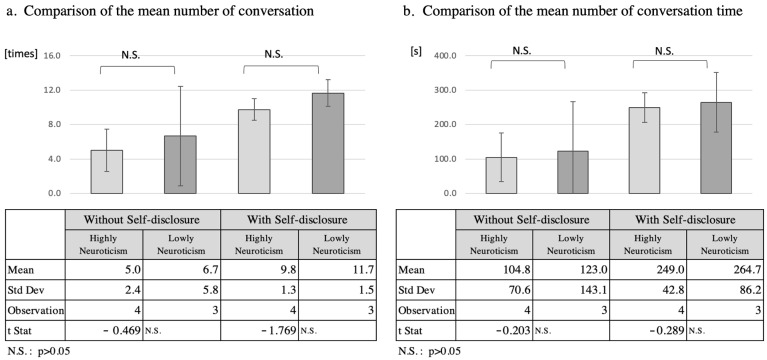
Comparison of number of conversation and speech duration according to high and low neuroticism.

As four of the seven participants had a neurotic tendency score of ≥3.5, and three participants scored less than 3.5, these two groups were compared as the high-neurotic group and the low-neurotic group (Figure 7). The mean number of interactions increased by 4.8 in the high-neurotic group from 5.0 to 9.8, while it increased by 5.0 in the low-neurotic group from 6.7 to 11.7. The total interaction time increased by 144.2 s from 104.8 s to 249.0 s in the high-neurotic tendency group, while in the low-neurotic tendency group it increased by 141.7 s from 123.0 s to 264.7 s. However, *t*-tests of the mean number of interactions and total interaction time for the two separate groups did not reveal a significant difference (*p* > 0.05).

#### 5.2.5. Analysis of Openness

**Figure 8:**
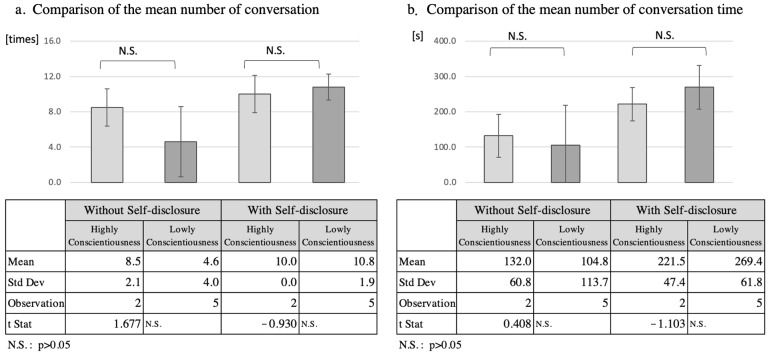
Comparison of number of conversation and speech duration according to high and low openness.

Since four of the seven participants had an openness score of ≥3.5, and three participants scored less than 3.5, these two groups were compared as high- and low-openness groups (Figure 8). The mean number of interactions increased by 7.0 in the high-openness group from 4.0 to 11.0, by 3.3, in the low-openness group from 7.0 10.3. The total interaction time increased by 165.0 s from 107.7 s to 272.7 s in the high-openness group, while in the low-openness group it increased by 126.7 s from 116.3 s to 243.0 s. However, *t*-tests of the mean number of interactions and total interaction time for the two separate groups did not reveal a significant difference (*p* > 0.05). Consequently, the mean number of interactions and the total interaction time for two separate groups did not reveal a significant difference in any participant’s personality traits in the Big Five model: extraversion, agreeableness, diligence, neuroticism, and openness.

## 6. Assessing the Validity of Speech Content by Its Emotional Value

Sentiment analysis involves the interpretation and classification of emotions (positive, negative, and neutral) in text data using natural language processing, text analysis, and computational linguistics to systematically identify, extract, quantify, and investigate emotional states and subjective information. Sentiment analysis tools allow for the analysis of large amounts of text data by quantifying them using emotional values.

### 6.1. Method

For sentiment analysis, we used the Japanese evaluation polarity dictionary developed by Inui et al. [53]. The Japanese Polarity Dictionary is a manually checked Japanese polarity dictionary with polarized (compound) nouns that includes approximately 8500 evaluative polarity expressions. The evaluation polarity has a value between −1.0 and 1.0, where −1.0 denotes the most negative polarity, 1.0 denotes the most positive polarity, and 0 means neutral polarity. Because all the recorded audio data were converted to text data in this study, sentiment analysis was performed to observe the emotional changes in the users’ speech.

### 6.2. Results

**Figure 9:**
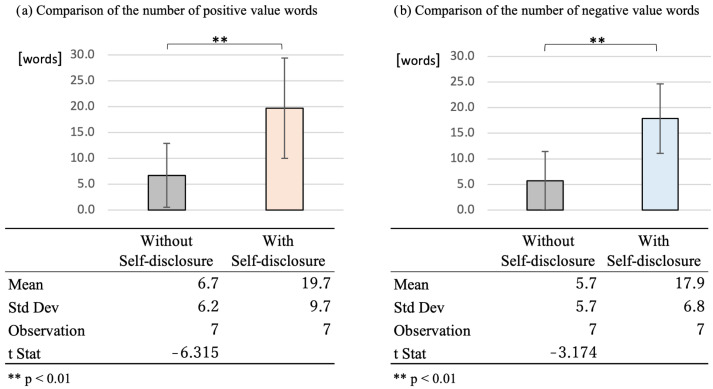
Supposed flow of participant cognition after the SAR self-disclosure.

Sentiment analysis revealed that the number of words with positive emotional values increased from an average of 6.7 words to 19.7 words per participant during the 10 days of the intervention (Figure 9). In addition, the number of words with negative emotional values increased from an average of 5.7 words to 17.9 words per participant during the 10 days of the intervention. Student’s *t*-tests were conducted for both the number of words with positive emotional values and the number of words with negative emotional values, under which the means in the same sample showed that both positive and negative words increased significantly before and after the intervention (*p* < 0.01).

## 7. Participant Interviews

Since data acquisition by each household is limited for privacy reasons, we analyzed the effects of self-disclosure by SARs in terms of quantitative aspects, such as the number of interactions, total interaction time, and positive and negative emotional values in previous chapters. However, because this intervention was carried out in each participant’s household, the effect of the intervention could not be completely understood via quantitative evaluation alone. Some studies have reported the effectiveness of group interviews for interventions involving a small number of participants. Therefore, a group interview with seven participants was conducted to determine how self-disclosure is perceived.

### 7.1. Method

Number of groups: 3 groups (2/2/3 participants per group); Group composition: participants, facilitators, and note-takers; Total interview time: 60 minutes per group. The seven participants were divided into three groups for the group interviews, and each group had an assigned facilitator and a note-taker in addition to the participants. As an interview procedure, the facilitator asked the participants questions, and note-takers recorded their opinions, while all voice data of the interviews were recorded. The facilitator gave the participants topics on the use case of the robot, changes in the first 10 days and the next 10 days, and changes in the participants’ perceptions of the robot. Participants were asked to speak freely about what they felt. After the group interviews, all recorded notes and audio data were analyzed.

### 7.2. Results

First, six of the seven participants made positive comments regarding the robot’s self-disclosure. However, four of the seven participants expressed negative opinions regarding the robot’s self-disclosure. Four of the seven participants felt that Papero was a human being, and they gave romantic advice to the robot or felt sorry about missing out on scheduling information support by the robot. Interestingly, three of the seven participants commented on their impressions of the robot. While two people personified the robot as “a young person,” another participant considered it “a young male.”

### 7.3. Analysis

**Figure 10:**
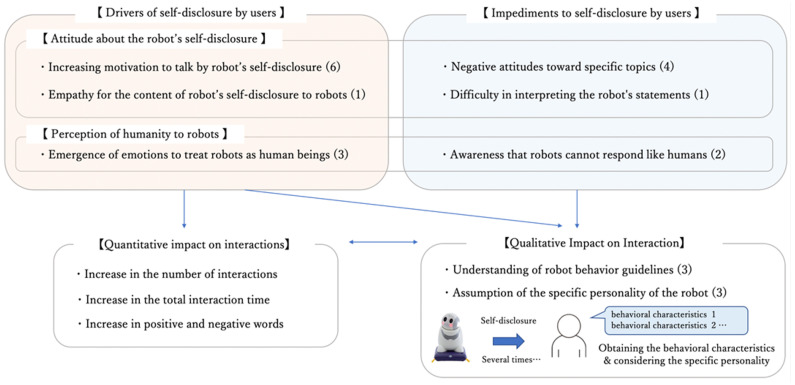
Comparison of the number of words appearing for each emotion value before and after the intervention.

Participants’ opinions were coded, and the concepts were summarized using the modified grounded theory approach (m-GTA) [54] (Figure 10). Based on the group interviews, the eight concepts coded were as follows: increasing motivation to talk by the robot’s self-disclosure (6), empathy toward robots due to the content of the robot’s self-disclosure (1), emergence of emotions to treat robots as human beings (3), negative attitudes toward specific topics (4), difficulty in interpreting the robot’s statements (1), awareness that robots cannot respond like humans (2), understanding of robot behavior guidelines (3), and assumption of the specific personality of the robot (3). The number inside ( ) indicates the number of people who shared that opinion during the group interviews. “Self-disclosure by the robot to increase the motivation to speak” (6), “empathy for self-disclosure to the robot” (1), and “the perception of humanity to the robot” (3) contributed to the user’s self-disclosure, which means an increase in the number of interactions, total interaction time, and positive and negative value words in the intervention. On the other hand, “negative attitudes toward certain topics (love and one’s own personality)” (4), “difficulty in interpreting the robot’s comments” (1), and “awareness that robots cannot respond like humans” (2) are factors that decrease users’ self-disclosure.

A previous study, “A Model of Personality Cognitive Processes by Self-Disclosure,” has shown that participants assume a specific personality when the agent discloses themselves several times to them. Based on these findings, it is considered that participants applied their mental model of others to robots based on comments such as “understanding the behavioral guidelines of robots” (3) and “assumption of the specific personality of the robot” (3). Using the grounded theory approach, we propose the following as a summary of the cognitive flow after self-disclosure by SAR: (1) first, participants observed self-disclosure by SAR and inferred the behavioral characteristics of SAR based on the self-disclosure contents; (2) participants observed self-disclosure by SAR several times and assumed more concrete behavioral guidelines for SAR; and (3) based on the accumulated behavioral guidelines, specific images such as the gender and age of the robot are considered by participants.

## 8. Discussion

### 8.1. Effectiveness of the Self-Disclosure Function

Considering the mean number of interactions, total interaction time, and results of sentiment analysis, robot self-disclosure is expected to be effective in improving the quantity and quality of interactions between older adults and robots. Although there were differences in cognitive function among the participants in this study and the effects of the intervention differed somewhat between the cognitively impaired and healthy older groups, the results suggest that the participants could communicate with the robot and that the proposed robot’s self-disclosure was effective if the MMSE score of participants was 25 or more. However, because the number of participants in this study was small (only seven people), studies with more participants are required.

### 8.2. Development of HRI Based on Self-Disclosure

The results of the sentiment analysis demonstrated that not only did the number of interactions and total time increase, but also the number of words with positive or negative values. Mutual self-disclosure may be triggered by SAR’s self-disclosure. In addition, since the self-disclosure intervention may change the interaction between SARs and users in terms of emotional values, the researcher may be able to remotely estimate the mental state of the participants based on the interactions and link them to social support, if necessary. In addition, this could be used for daily monitoring of older users and remote healthcare in rural areas with insufficient medical resources. Furthermore, because participants’ interest areas are revealed by their sentiment values, data mining may allow researchers to recommend topics of greater interest to them, similar to the history of an internet browser.

### 8.3. Improvements to the Self-Disclosure Script

As for the group interviews, the self-disclosure script required some improvements based on negative feedback. First, there were three comments that they had confused themselves with specific questions, such as the story of first love. As self-disclosure has a scale of depth, it is necessary to consider the scale and arrange the order of self-disclosure according to the participants. Second, there were three comments about the structure of the questioning after self-disclosure and difficulty in understanding the intent of the conversation; however, improvement was expected by adjusting the items based on the feedback of participants.

In this study, a blog corpus was used to formulate speech content, and one of the promising approaches is to work with users to create the robot’s speech content. However, as Sanders et al. report, co-creation and design is performed differently in the U.S. and Europe, and may be culturally different in Asia as well [55]. Further research should clarify which content is preferred by which cultures.

### 8.4. The Reason That Participants Assumed a Relatively Young Generation for SARS

The self-disclosure intervention revealed that the participants assumed a relatively young personality for SARs. This may be because the KNB corpus, which forms the basis of the robot’s self-disclosure script, was written by 81 Japanese university students. The results suggest that participants’ perception of SARs can be changed arbitrarily by the speech script, regardless of the appearance of the SAR, although further study is required.

## 9. Limitations

It is not clear in the negative responses to the robot’s self-disclosure, whether the participants are already prejudiced against robots, or simply do not like them. In addition, how the character of the robot is recognized by the participants is beyond the scope of this study, and further research needs to be conducted separately.

## 10. Conclusions

In this study, we defined the specific requirements and functions of self-disclosure in SARs and developed ten non-arbitrary speech scripts from the field of social psychology using the Japanese corpus and self-disclosure items. To evaluate the effect of self-disclosure in SARs, an SAR was introduced into the households of seven community-dwelling older adults for 20 days with their consent. The 20 days were divided into pre- and post-test comparisons, with the first 10 days without self-disclosure and the last 10 days with self-disclosure. All spoken dialogues between older adults and the robot were recorded. Based on the recorded spoken dialogue data, we analyzed how SAR’s self-disclosure changed the number of verbal dialogues, the total duration of the dialogues, and the quality of the dialogues. We also checked whether the effects differed depending on the cognitive function and personality characteristics of the participants.

We analyzed how the number of verbal interactions, total time of the interactions, and quality of the interactions changed by SAR’s self-disclosure from recorded voice interaction data. As a result, during the 20 days of intervention, (a) the mean number of interactions was 5.7 (SD = 3.9) in the first 10 days, but increased to 10.6 (SD = 1.6) in the next 10 days with SAR’s self-disclosure. As for (b), the mean total interaction time was 112.6 s (SD = 97.0) in the first 10 days, increasing to 255.7 s (SD = 58.9) in the next 10 days with the SAR’s self-disclosure. There were significant differences in the number and duration of interactions before and after the intervention (*p* < 0.01).

In addition, as individual differences are particularly important when social robots are used, the effect of self-disclosure by SAR was assessed according to the cognitive function and personality traits of individual users. With regard to cognitive function, the mean number of interventions and the total interaction time were higher in the healthy older group when viewed as a percentage increase. However, a *t*-test of means, assuming two separate populations, did not reveal a significant difference (*p* > 0.05). As for the participants’ personality traits, the mean number of interactions and the total interaction time for the two separate groups did not reveal a significant difference in any participant’s personality traits in the Big Five model: extraversion, agreeableness, diligence, neuroticism, and openness. Furthermore, a sentiment analysis of the user’s speech revealed that the number of words with positive emotional values increased from an average of 6.7 words to 19.7 words per participant during the 10 days of the intervention. In addition, the number of words with negative emotional values increased from an average of 5.7 words to 17.9 words per participant during the 10 days of the intervention. Student’s *t*-tests of the same sample demonstrated that the number of both positive and negative value words increased significantly from before to after the intervention (*p* < 0.01).

Finally, we conducted group interviews with the participants and received positive comments regarding the robot’s self-disclosure. Some participants considered the specific personality of the SAR by accumulating its behavioral characteristics. These results indicate that the robot’s self-disclosure feature is effective in significantly increasing the quantity and quality of verbal interactions with older adults.

## Figures and Tables

**Table 1:** Self-disclosure items and their categories.

No.	KNB Corpus Contents	Category of Self-Disclosure Items
1	News that is on my mind lately	Attitudes and Opinions
2	Favorite food/frequently visited shops	Interests
3	Favorite music	Interests
4	Current goals you have	Work
5	Uses for luxury	Money
6	An embarrassing incident	Interpersonal relationships
7	Guilt-tripping incidents	Interpersonal relationships
8	Own personality that needed to improve	Interpersonal relationships
9	Memories of love	Interpersonal relationships
10	Efforts for health	Body

**Table 2:** Number of interactions and interaction time during the 20 days of intervention.

				Number of Interactions (Times)		Interaction Time (s)	
No.	Age	Gender	MMSE	Before Intervention	After Intervention	Before Intervention	After Intervention
1	76	Male	27	0	12	0	248
2	69	Male	27	10	13	280	358
3	69	Female	26	4	10	45	222
4	75	Female	28	10	10	89	188
5	76	Female	26	7	10	175	255
6	76	Male	30	7	11	156	307
7	62	Female	30	2	8	43	212
Avg.	71.9	NA	27.7	5.7	10.6	112.6	255.7

## Data Availability

The data that support the findings of this study are available from the corresponding author upon reasonable request.

## References

[1] Ministry of Health, Labor and Welfare (2022). Annual Report for Aging Society.

[2] Tokyo Metropolitan Government Bureau of Social Welfare and Public Health (2010). 2010 Basic Survey on Welfare and Public Health in Tokyo.

[3] Nihei M., Inoue T., Nishiura Y., Mamiya I., Onaka S., Watabe K., Osawa Y., Shimizu Y., Harada A., Hiroaki K. (2015). Relaxed watching system for elderly by using communication robot. JSMBE.

[4] Chen S.C., Jones C., Moyle W. (2018). Social Robots for Depression in Older Adults: A Systematic Review. Nurs. Scholarsh..

[5] Pino M., Boulay M., Jouen F., Rigaud A.-S. (2015). “Are we ready for robots that care for us?” Attitudes and opinions of older adults toward socially assistive robots. Front. Aging Neurosci..

[6] Vandemeulebroucke T., De Casterlé B.D., Gastmans C. (2018). How do older adults experience and perceive socially assistive robots in aged care: A systematic review of qualitative evidence. Aging Ment Health.

[7] Góngora Alonso S., Hamrioui S., de la Torre Díez I., Motta Cruz E., López-Coronado M., Franco M. (2019). Social Robots for People with Aging and Dementia: A Systematic Review of Literature. Telemed. e-Health.

[8] Vandemeulebroucke T., de Casterlé B.D., Gastmans C. (2018). The use of care robots in aged care: A systematic review of argument-based ethics literature. Arch. Gerontol. Geriatr..

[9] Takama Y., Namba H., Iwase N., Hattori S., Muto Y., Shoji T. Human-Robot Communication Based on Information Recommendation during TV Viewing. Proceedings of the National Conference on Artificial Intelligence.

[10] Derlega V.J., Metts S., Petronio S., Margulis S.T. (1993). Self-Disclosure.

[11] Joanne F. (2006). Experimental disclosure and its moderators: A meta-analysis. Psychol. Bull..

[12] Pennebaker J.W., Graybeal A. (2001). Patterns of natural language use: Disclosure, personality, and social integration. Curr. Dir. Psychol. Sci..

[13] Trepte S., Scharkow M., Reinecke L., Oliver M.B. (2016). Friends and lifesavers: How social capital and social support received in media environments contribute to well-being. The Routledge Handbook of Media Use and Well-Being: International Perspectives on Theory and Research on Positive Media Effects.

[14] House J.S. (1981). Work Stress and Social Support.

[15] Collins N.L., Miller L. (1994). Self-disclosure and liking: A meta-analytic review. Psychol. Bull..

[16] Ikeda K., Bada I., Hodari K. (2018). A communication support system by encouraging self-disclosure. Interaction 2018 Proceedings.

[17] Feil-Seifer D., Mataric M.J. Defining Socially Assistive Robotics. Proceedings of the 9th International Conference on Rehabilitation Robotics (ICORR 2005).

[18] Kazuhiko M. From Attractive Robots to Persuasive Robots. Proceedings of the 24th National Conference of Japanese Society for Artificial Intelligence, 2J1-OS6-9.

[19] Yamamoto D., Doi M., Matsuhira N., Ueda H., Kidode M. Familiar Behaviors Evaluation for a Robotic Interface of Practicality and Familiarity. Proceedings of the 4th IEEE International Conference on Development and Learning (ICDL-05).

[20] Banks M.R., Willoughby L.M., Banks W.A. (2008). Animal-Assisted Therapy and Loneliness in Nursing Homes: Use of Robotic versus Living Dogs. J. Am. Med. Dir. Assoc..

[21] Shibata T., Mitsui T., Wada K., Touda A. Mental Commit Robot and its Application to Therapy of Children. Proceedings of the IEEE/ASME International Conference on Advanced Intelligent Mechatronics Proceedings.

[22] Ninomiya T. (2015). Introduction of Communication Robot “PALRO" and its Activities in Sagami Robot Industry Special Zone. J. Robot. Soc. Jpn..

[23] LOVOT, GROOVE X, Inc.. https://lovot.life/.

[24] Aibo, Sony. https://aibo.sony.jp/.

[25] Moyle W., Cooke M., Beattie E., Jones C., Klein B., Cook G., Gray C. (2013). Exploring the effect of companion robots on emotional expression in older adults with dementia: A pilot randomized controlled trial. J. Gerontol. Nursing.

[26] Robinson H., MacDonald B., Kerse N., Broadbent E. (2013). The psychosocial effects of a companion robot: A randomized controlled trial. J. Am. Med. Dir. Assoc..

[27] Nakagawa K., Shinozawa K., Matsumura R., Ishiguro H., Hagita N. (2007). Persuasion effect by adding personality to a health care robot. Proc. Forum Inf. Sci. Technol..

[28] Ogawa Y., Miyazawa Y., Kikuchi H. (2013). Giving Personality to Spoken Dialogue Agents by Self-Disclosure. Trans. Hum. Interface Soc..

[29] Kang S.-H., Gratch J. (2010). Virtual humans elicit socially anxious interactants’ verbal self-disclosure. Comput. Animat. Virtual Worlds.

[30] Kumazaki H., Warren Z., Swanson A., Yoshikawa Y., Matsumoto Y., Takahashi H., Sarkar N., Ishiguro H., Mimura M., Minabe Y. (2018). Can Robotic Systems Promote Self-Disclosure in Adolescents with Autism Spectrum Disorder? A Pilot Study. Front. Psychiatry.

[31] Noguchi Y., Kamide H., Tanaka F. (2020). Personality Traits for a Social Mediator Robot Encouraging Elderly Self-Disclosure on Loss Experiences. ACM Trans. Hum.-Robot Interact..

[32] Hashimoto R., Kurohashi S., Kawahara D., Niizato K., Nagata M. (2011). Construction of a blog corpus with syntactic, matching, and evaluation information. Nat. Lang. Process..

[33] Jourard S.M. (1964). The Transparent Self: Self-Disclosure and Well-Being.

[34] Inoue T., Nihei M., Narita T., Onoda M., Ishiwata R., Mamiya I., Motoki S., Hiroaki K., Shinichi O., Yoshihiro F. (2012). Field-based development of an information support robot for persons with dementia. Technol. Disabil..

[35] Kaufer D.I., Williams C.S., Braaten A.J., Gill K., Zimmerman S., Sloane P.D. (2008). Cognitive Screening for Dementia and Mild Cognitive Impairment in Assisted Living: Comparison of 3 Tests. J. Am. Med. Dir. Assoc..

[36] Saxton J., Morrow L., Eschman A., Archer G., Luther J., Zuccolotto A. (2009). Computer Assessment of Mild Cognitive Impairment. Postgrad. Med..

[37] Folstein M.F., Folstein S.E., McHugh P.R. (1975). Mini-Mental State: A practical method for grading the cognitive state of patients for the clinician. Psychiatr. Res..

[38] Tsoi K.K.F., Chan J.Y.C., Hirai H.W., Wong S.Y.S., Kwok T.C.Y. (2015). Cognitive Tests to Detect Dementia: A Systematic Review and Meta-Analysis. JAMA Intern. Med..

[39] https://www.necplatforms.co.jp/solution/papero_i/index.html.

[40] Riek L.D., Luxton D.D. (2016). Robotics technology in mental health care. Artificial Intelligence in Behavioral and Mental Health Care.

[41] Aly A., Tapus A. A model for synthesizing a combined verbal and nonverbal behavior based on personality traits in human-robot interaction. Proceedings of the 8th ACM/IEEE International Conference on Human-Robot Interaction.

[42] Andrist S., Mutlu B., Tapus A. (2015). Look like me: Matching robot personality via gaze to increase motivation. Proceedings of the 33rd Annual ACM Conference on Human Factors in Computing Systems.

[43] Lee K.M., Nass C. (2003). Designing social presence of social actors in human computer interaction. Proceedings of the SIGCHI Conference on Human Factors in Computing Systems.

[44] Mileounis A., Cuijpers R.H., Barakova E.I. (2015). Creating robots with personality: The effect of personality on social intelligence. Artificial Computation in Biology and Medicine.

[45] Tapus A., Matarić M.J. Socially Assistive Robots: The Link between Personality, Empathy, Physiological Signals, and Task Performance. Proceedings of the AAAI Spring Symposium on Emotion, Personality, and Social Behavior.

[46] John O.P., Srivastava S., Pervin L.A., John O.P. (1999). The big five trait taxonomy: History, measurement, and theoretical perspectives. Handbook of Personality, Second Edition: Theory and Research.

[47] Nass C., Lee K.M. (2001). Does computer-synthesized speech manifest personality? Experimental tests of recognition, similarity-attraction, and consistency-attraction. J. Exp. Psychol. Appl..

[48] Nass C., Moon Y., Fogg B.J., Reeves B., Dryer D.C. (1995). Can computer personalities be human personalities?. Int. J. Hum.-Comput. Stud..

[49] Cuperman R., Ickes W. (2009). Big Five predictors of behavior and perceptions in initial dyadic interactions: Personality similarity helps extraverts and introverts, but hurts “disagreeables”. J. Pers. Soc. Psychol..

[50] Amirazodi F., Amirazodi M. (2011). Personality traits and Self-esteem. Procedia-Soc. Behav. Sci..

[51] Kernis M.H. (2003). Target article: Toward a Conceptualization of Optimal Self-Esteem. Psychol. Inq..

[52] Oshio A., Shingo A.B.E., Cutrone P. (2012). Development, reliability, and validity of the Japanese version of Ten Item Personality Inventory (TIPI-J). Jpn. J. Personal..

[53] Higashiyama M., Inui K., Matsumoto Y. (2008). Learning Sentiment of Nouns from Selectional Preferences of Verbs and Adjectives. Proceedings of the 14th Annual Meeting of the Association for Natural Language Processing.

[54] Yasuhito K. (2007). Analytical Techniques of the Modified Grounded Theory Approach (M-GTA). J. Univ. Toyama Nurs. Soc..

[55] Sanders E.B.-N., Stappers P.J. (2008). Co-creation and the new landscapes of design. CoDesign.

